# Bmi-1 directly upregulates glucose transporter 1 in human gastric adenocarcinoma

**DOI:** 10.1515/biol-2022-0024

**Published:** 2022-03-24

**Authors:** Ying Guo, Guangyu Zhou, Qingjie Ma, Li Zhang, Jiwei Chen

**Affiliations:** Department of Nephrology, China-Japan Union Hospital, Jilin University, Changchun, Jilin 130033, China; Department of Nuclear Medicine, China-Japan Union Hospital, Jilin University, 126 Xiantai St., Changchun, Jilin 130033, China; Department of Neurology, China-Japan Union Hospital, Jilin University, 126 Xiantai St., Changchun, Jilin 130033, China

**Keywords:** gastric adenocarcinoma, glucose metabolism, glucose transporter 1, Bmi-1, ^18^F-FDG uptake, PET/CT imaging

## Abstract

This study aimed to investigate whether and how Moloney murine leukemia virus integration site 1 (Bmi-1) plays a role in the regulation of glucose transporter 1 (GLUT1) in gastric adenocarcinoma (GAC). GAC and matched noncancerous tissues were obtained from GAC patients who underwent surgical treatment at the China-Japan Union Hospital, Jilin University (Changchun, Jilin, China). The human GAC cell line AGS and the gastric epithelial cell line GES-1 were used for *in vitro* studies. BALB/c nude mice were used for *in vivo* studies. The Bmi-1 and GLUT1 protein levels were significantly greater in human tissues from GAC patients and AGS cells in comparison with controls. Silencing of Bmi-1 resulted in significant decrease in glucose uptake, lactate levels, and GLUT1 expression. *In vivo* ^18^F-deoxyglucose positron emission tomography/computed tomography (^18^F-FDG PET/CT) imaging studies indicated that the nude mice bearing xenografts of AGS cells treated with Bmi-1-specific small interfering RNA (siRNA) had a significantly lower maximum standardized uptake value (SUV_max_) in comparison with the control mice. Thus, Bmi-1 directly upregulates GLUT1 gene expression, through which it is involved in enhancing glucose uptake in GAC. The results also provide scientific evidence for ^18^F-FDG PET/CT imaging to evaluate Bmi-1 and glucose uptake in GAC.

## 1 Introduction

Gastric adenocarcinoma (GAC), a predominant form of gastric cancer, is among the most common malignancies worldwide. The incidence of GAC is particularly high in China and some other countries in East Asia [1]. In China, approximately 679,000 individuals were newly diagnosed with gastric cancer and nearly 498,000 gastric cancer patients died in 2015 [2]. Notably, the majority of gastric cancer cases are detected and diagnosed at a late stage, when the disease has progressed into advanced cancer; and the 5-year survival rate has been reported to be as low as 20–25% [3,4]. Therefore, it is critical to understand the mechanisms underlying the carcinogenesis and progression of gastric cancer, which may guide the development of new therapeutic approaches.

It has been well documented that during the abnormally rapid growth of cancer cells, glucose uptake and metabolism are markedly enhanced to meet the increased need for energy [5,6]. Warburg found that cancer cells uptake glucose preferably for glycolysis even under aerobic conditions with sufficient oxygen, which is referred to as the Warburg effect [5,6].

Glucose transporter 1 (GLUT1) has been shown to play a critical role in glucose uptake, a rate-limiting step in glucose metabolism[7,8]. However, it remains largely unknown how GLUT1 is regulated to facilitate glucose uptake in cancer cells, including GAC cells. More recently, we identified a significant correlation between GLUT1 and Moloney murine leukemia virus integration site 1 (Bmi-1) in gastric cancer (unpublished data, this study has been submitted but has not been published yet).

A number of previous studies have demonstrated that Bmi-1 is involved in multiple cellular processes to promote the proliferation, invasion, and metastasis of gastric cancer; and its expression is positively correlated with the malignancy of gastric cancer [9,10,11,12]. Recently, we found that the expression of Bmi-1 was significantly greater in GAC tissues than in the matched histologically cancer-free specimens (unpublished data). We also identified a positive correlation between a higher expression of Bmi-1 and more advanced clinical stages, according to the clinicopathologic classifications of patients with gastric cancer. More recently, we noticed that there was a significant correlation between GLUT1 expression and Bmi-1 expression in gastric cancer (unpublished data).

Intrigued by and building upon these previous findings, we aimed to further investigate whether and how the transcription factor Bmi-1 plays a role in the regulation of GLUT1 in GAC. The resulting data may provide scientific evidence supporting Bmi-1 as a novel target for the development of new therapies for gastric cancer and as a new biomarker to be evaluated by the maximum standardized uptake value (SUV_max_) using ^18^F-deoxyglucose positron emission tomography/computed tomography (^18^F-FDG PET/CT) imaging.

## 2 Materials and methods

### 2.1 Human tissue samples

Four paired GAC and control tissues were obtained from GAC patients who underwent surgical treatment at the China-Japan Union Hospital, Jilin University (Changchun, Jilin, China). The characteristics of the patients are shown in Table 1. The diagnosis of GAC was histopathologically made and confirmed by pathologists at the Department of Pathology, the China-Japan Union Hospital, Jilin University. The matched control tissues were histologically normal paracancerous tissues.

**Table 1:** Characteristics of the patients with gastric adenocarcinoma

Characteristics	Patient 1	Patient 2	Patient 3	Patient 4
Age (years)	69	48	66	34
Sex	Male	Male	Female	Female
Tumor diameter (cm)	<5	<5	≥5	<5
Degree of pathological differentiation	PD	PD	PD	MD
T stage	T3/T4	T3/T4	T1/T2	T3/T4
N stage	N3	N3	N1	N3
Lauren’s classification	IT	Diffuse/Mixed type	IT	Diffuse/Mixed type

Note: PD, poorly-differentiated; MD, moderately-differentiated; IT, intestinal type; DT, diffuse type.


**Informed consent:** Informed consent has been obtained from all individuals included in this study.
**Ethical approval:** The research related to human use has been complied with all the relevant national regulations, institutional policies, and in accordance with the tenets of the Helsinki Declaration, and has been approved by the Ethics Committee, equivalent to the institutional review board, at the China-Japan Union Hospital, Jilin University.

### 2.2 Experimental animals

The 4–6-week-old BALB/c nude mice used in this study were purchased from Nanjing Biomedical Research Institute (Nanjing, Jiangsu, China) and maintained in a pathogen-free environment with constant temperature and humidity at the Animal Center of Jilin University (Changchun, Jilin, China). The bedding was regularly changed for the experimental mice, and they had access to adequate water.


**Ethical approval:** The research related to animal use has been complied with all the relevant national regulations and institutional policies for the care and use of animals and was approved by the Ethics Committee of Jilin University (Changchun, Jilin, China).

### 2.3 Cell culture and transfection

Two cell lines, including human GAC AGS cells and gastric epithelial GES-1 cells, were obtained from Shanghai Zhongqiao Xinzhou Biotechnology Co., Ltd (Shanghai, China) and were used in this study. The AGS cell line was derived from the gastric tissue of a patient suffering from gastric cancer, while the noncancerous GES-1 cell line originated from primary fetal gastric epithelial cells infected with simian vacuolating virus 40. The cells were maintained in RPMI-1640 basal cell culture medium supplemented with 10% fetal bovine serum (Hyclone, American) and 1% penicillin/streptomycin in a CO_2_ incubator at 37°C and 5% CO_2_. For transfection, the AGS cells were seeded in a 24-well plate. When the AGS cells reached approximately 70% confluency, they were transfected with Bmi-1-specific small interfering RNA (siRNA) (Cell Signaling Technology, USA) in the experimental group or nonspecific control siRNA (Cell Signaling Technology, USA) in the control group using the transfection reagent Lipofectamine 2000 (Invitrogen, USA), according to the manufacturer’s instructions. At 48 h after transfection, the cells were harvested for subsequent analysis.

### 2.4 Immunohistochemistry

Immunohistochemistry was conducted to examine the Bmi-1 and GLUT1 protein expression levels in GAC and paired histologically noncancerous tissues from patients who underwent surgical resection of GAC. Horseradish peroxidase (HRP)-conjugated goat anti-rabbit IgG (dilution, 1:500; Jackson ImmunoResearch), Bmi-1 primary antibody (dilution, 1:500; Bioss Antibodies, Beijing, China), and GLUT1 primary antibody (dilution, 1:1,000; Affinity Biosciences, Cincinnati, OH, USA) were used in the immunohistochemical analysis according to the standard protocol in our laboratory.

### 2.5 Western blot analysis of Bmi-1 and GLUT1 protein expression

Western blot analysis was performed to determine the Bmi-1 and GLUT1 protein expression in AGS cells. In brief, total proteins were extracted from the cell pellets by adding ice-cold lysis buffer (Wanleibio, Shenyang, China) containing the protease inhibitor phenylmethylsulfonyl fluoride and phosphatase inhibitor at a 1:100 dilution in lysis buffer. After incubation on ice for 30 min, the mixture was centrifuged at 12,000 rpm and 4°C for 20 min, and the total proteins of the cell lysates in the supernatant were collected and quantified using the bicinchoninic acid assay. The total protein sample was loaded onto 10–12% sodium dodecyl sulfate–polyacrylamide gels and subjected to electrophoresis. After electrophoretic transfer onto a polyvinylidene fluoride membrane (IPVH00010, Millipore, USA), the resulting membrane was blocked with 5% nonfat milk and then incubated with specific primary antibodies, including Bmi-1 primary antibody (dilution, 1:400; bs-2999R Bioassen, Beijing, China), GLUT1 primary antibody (dilution, 1:1,000; AF0173, Affinity Biosciences, Cincinnati, OH, USA), and glyceraldehyde-3-phosphate dehydrogenase (GAPDH) primary antibody (WL01845, Wanleibio, Shenyang, China), respectively, at 4°C overnight. After washing at least three times with tris-buffered saline containing Tween 20, the membrane was incubated with goat anti-rabbit IgG-HRP secondary antibody (dilution, 1:5,000; 111-035-003, Jackson ImmunoResearch, USA). Enhanced chemiluminescence reagent was purchased from Wanleibio (WLA003, Shenyang, China) and used for visualization of the protein bands. A gel image processing system and Gel-Pro-Analyzer software were used for densitometric analysis of each specific protein band obtained by western blot analysis.

### 2.6 Quantitative real-time polymerase chain reaction (qPCR) analysis of GLUT1 mRNA

Total RNA was extracted from treated cells, and cDNA was synthesized. qPCR was performed using a real-time qRT-PCR kit (Takara, Japan), according to the manufacturer’s instructions. The primers used in the qPCR analysis were as follows: GLUT1, forward 5′-TCGTCGGCATCCTCATCGCC-3′ and reverse 5′-CCGGTTCTCCTCGTTGCGGT-3′ and GAPDH, forward 5′-AATCCCATCACCATCTTCCAC-3′ and reverse 5′-TGGACTCCACGACGTACTCA-3′.

### 2.7 Measurement of glucose uptake and lactate levels

The effect of Bmi-1 on glucose uptake in AGS cells was measured using a Glucose Uptake Assay Kit (Colorimetric, Abcam, ab136955, Cambridge, MA, USA), according to the manufacturer’s protocol. In brief, 10 µL of 2-deoxyglucose was added to AGS cells and incubated at 37°C for 20 min. Following incubation, the AGS cells were washed with phosphate-buffered saline three times and lysed with extraction buffer. The lysate was heated at 85°C for 40 min and then placed on ice for 5 min. The samples were mixed with reaction mix A, followed by reaction mix B. The absorbance was read at 412 nm on a BioTek microplate reader.

The lactate levels were examined using an L-Lactate Assay kit (colorimetric, Abcam, ab65331, Cambridge, MA, USA), according to the manufacturer’s instructions. Briefly, the samples were incubated with reaction mix for 30 min at room temperature and then read at 450 nm using a BioTek microplate reader.

### 2.8 Chromatin immunoprecipitation (ChIP) and ChIP-qPCR assays

The interaction between the transcription factor Bmi-1 and the promoter region of the GLUT1 DNA was examined by using a ChIP Assay Kit (WLA106a, Wanleibio, Shenyang, China). In brief, AGS cells were incubated with 1% formaldehyde at 37°C for 10 min, and the cross-linking reaction was terminated by the addition of 125 mM glycine and incubation for another 10 min. The AGS cells were pelleted, washed with phosphate-buffered saline, and lysed with cell lysis buffer. The resulting cell lysates were pelleted, washed, resuspended, and sonicated to break chromatin into DNA fragments. The resulting extracts were incubated with 2 μg Bmi-1 antibody (A0211, ABclonal, Wuhan, China) in the experimental group or with 1 μg control IgG in the negative control group at 4°C overnight. The immunocomplexes were precipitated with Protein G agarose beads, washed, eluted, and disassociated. The DNA disassociated with the immunocomplexes (protein-DNA complexes) was purified using a Gel Extraction and Purification Kit (WLA052a, Wanleibio, Shenyang, China). The purified DNA sample was then used as a template in the qPCR to amplify the target DNA using 2× Power Taq PCR MasterMix (PR1702, BioTeke, Beijing, China). The primers used for the qPCR in the ChIP assay were synthesized by Genscript Technology (Nanjing, China).

Following ChIP analysis, qPCR was carried out to amplify and quantify the GLUT1 promoter regions in the experimental group (Bmi-1-specific antibody) and negative control (IgG). The sequences of the primers were as follows: GLUT1, forward primer 5ʹ-TGAGCACGCCAGGGAGCAGG-3ʹ and reverse primer 5ʹ-TGGCTCTGGCTGCGCCGGGTAC-3ʹ.

### 2.9 BALB/c nude mice bearing human GAC AGS cells

For the *in vivo* studies, 10 BALB/c nude mice (age: 4–6 weeks; 6 males and 4 females) were used to generate human GAC xenografts. The BALB/c nude mice were randomly assigned into two groups. The nude mice were subcutaneously injected with 0.2–0.3 mL (5 × 10^6^ cells per mouse) of AGS cells treated with Bmi-1-specific siRNA in the experimental group (*n* = 5, 3 males and 2 females), or cells treated with nonspecific siRNA as a control (NC) in the control group (*n* = 5, 3 males and 2 females). The maximum tumor size was smaller than 1.5 cm in diameter.

### 2.10 ^18^F-FDG PET/CT imaging

^18^F-FDG PET/CT imaging was performed using the SuperArgus PET/CT imaging system for small-to-medium animals (Sedecal, Spain), as described previously [13,14]. Before imaging, the mice in the experimental and control groups were fasted for at least 6 h. ^18^F-FDG was diluted in normal saline and was injected into the tail vein at a dosage of 200 microcuries (μCi) (Radioactivity Meter, Capintec, USA). The mice were anesthetized with 4% isoflurane using an animal anesthesia machine (Midmark, USA) and an isoflurane vaporizer (Midmark, USA). After approximately 60 min, PET/CT imaging was performed on the mice. During the entire PET/CT imaging process, the mice were given 2.5% isoflurane to maintain anesthetization. Imaging data of the PET/CT scans were collected and subsequently analyzed using PMOD software (PMOD Technology, Switzerland).

### 2.11 Statistical analysis

Quantitative data were expressed as the mean value ± standard deviation. Statistical analysis was conducted using SPSS19.0 software. For multiple comparisons, one-way analysis of variance with *post-hoc* Tukey’s honestly significant difference test was used to analyze the difference between groups. *P* < 0.05 was considered a significant difference between groups.

## 3 Results

### 3.1 Bmi-1 and GLUT1 protein expression levels in GAC and paired noncancerous tissues

**Figure 1:**
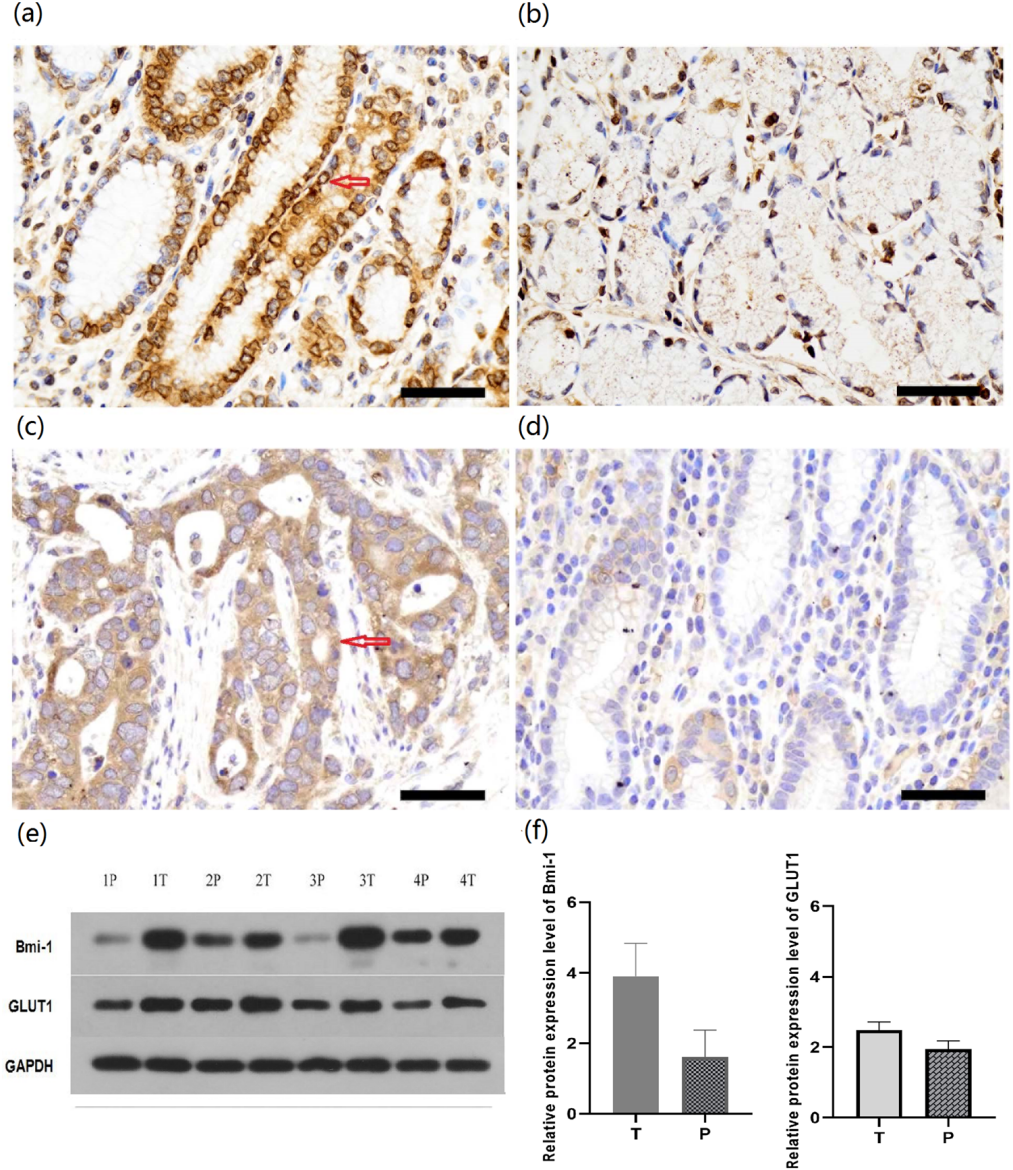
Bmi-1 and GLUT1 protein expression levels in (GAC) tissues and normal gastric specimens. Immunohistochemistry and western blot analysis were performed to examine the Bmi-1 and GLUT1 protein expression levels in GAC specimens (*n* = 4) and matched histologically normal gastric specimens (*n* = 4). Representative images of immunohistochemical staining of Bmi-1 in (a) GAC and (b) noncancerous control tissues (magnification, 400×). The yellow-brown color denotes positive Bmi-1 expression that is mainly located in the nucleus (arrows). Representative images of immunohistochemical staining of GLUT1 in (c) GAC and (d) noncancerous control tissues (magnification, 400×). GLUT1 is mainly localized in the cell membrane (arrows); (e) Western blot analysis of Bmi-1 and GLUT1 protein expressions in four paired tissues of GAC and noncancerous control tissues. The relative Bmi-1 and GLUT1 protein levels were normalized to GAPDH; (f) quantification of the Bmi-1 and GLUT1 protein expression levels in three independent experiments on the basis of the immunohistochemical and western blot images. The levels of Bmi-1 and GLUT1 were significantly greater in AGC tissues vs matched adjacent noncancerous tissues (3.903 ± 0.9363 vs 1.613 ± 0.760 and 2.480 ± 1.276 vs 1.945 ± 0.230, respectively) (All *P* < 0.05). T, tumor tissues; P, paracancerous, noncancerous tissues.

We initially examined the Bmi-1 and GLUT1 protein expressions in GAC and matched histologically noncancerous tissues in patients with GAC. Representative images of immunohistochemistry assays revealed that the Bmi-1 and GLUT1 protein expression levels were abnormally elevated in GAC compared with the matched histologically noncancerous tissues (Figure 1a–d). The western blot analysis data were in agreement with those of the immunohistochemical examinations (Figure 1e–f). Both the immunohistochemistry and western blot analyses indicated that the Bmi-1 and GLUT1 protein expression levels were markedly increased in GAC compared with the paired noncancerous tissues.

### 3.2 Effects of Bmi-1 on glucose uptake and lactate levels in AGS cells

**Figure 2:**
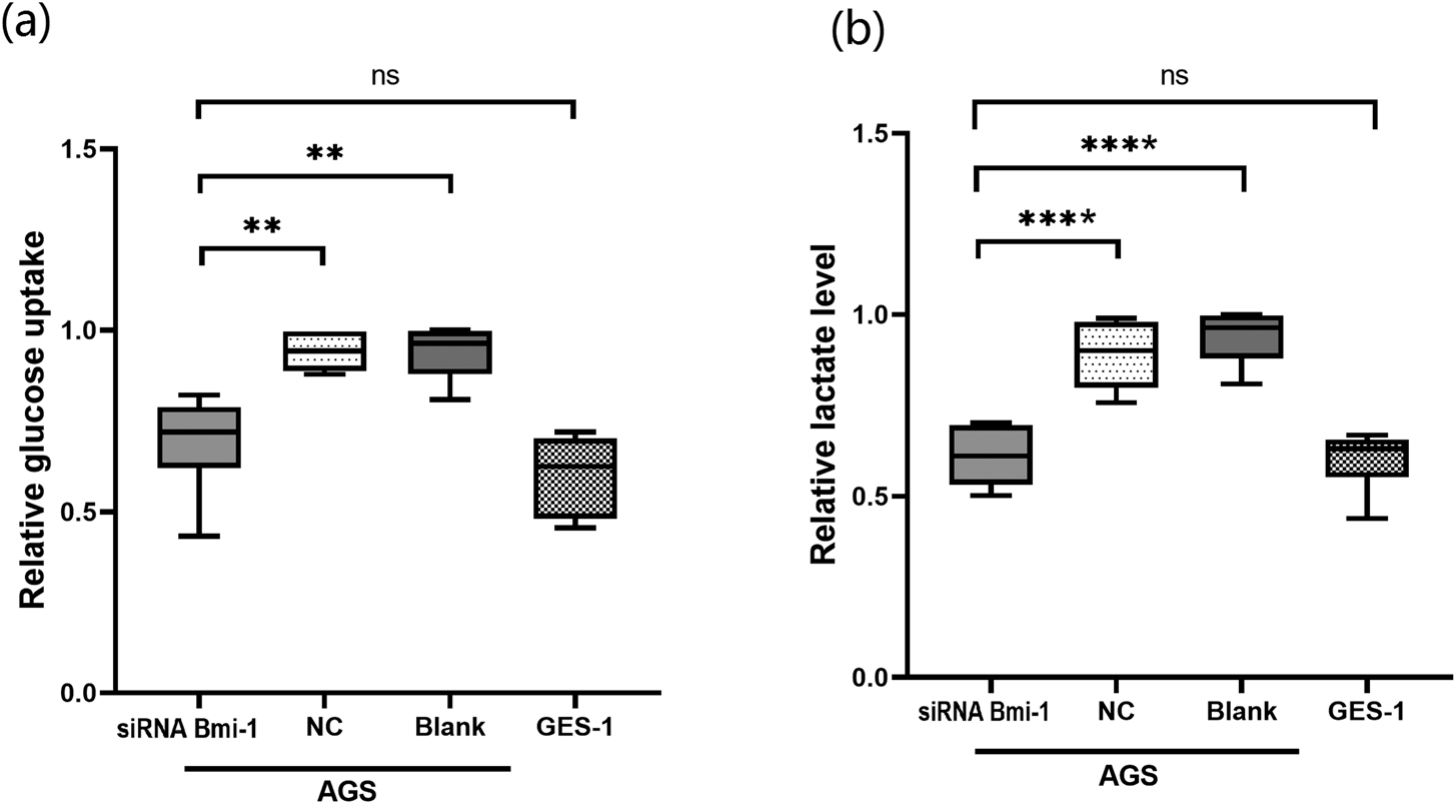
Effects of silencing the Bmi-1 gene on glucose uptake and lactate levels in human gastric adenocarcinoma AGS cells. (a) Relative glucose uptake in AGS cells transfected with Bmi-1-specific siRNA (Bmi-1 siRNA), nonspecific siRNA as a control (NC), and without any treatment (Blank) as well as in human immortalized gastric epithelial GES-1 cells. The relative glucose uptake was significantly decreased in AGS cells transfected with Bmi-1 siRNA vs NC. ** indicates a significant difference between the two groups, *P* < 0.05; (b) relative lactate levels in AGS cells transfected with Bmi-1 siRNA, NC, and without any treatment (Blank), and in GES-1 cells. The relative lactate level was significantly lower in AGS cells transfected with Bmi-1 siRNA vs NC. ** indicates a significant difference between the two groups, *P* < 0.05.

To investigate the potential role of Bmi-1 in glucose metabolism in GAC, the Bmi-1 gene expression was downregulated in AGS cells by Bmi-1-specific siRNA. As shown in Figure 2a, the relative glucose uptake was significantly decreased in AGS cells transfected with Bmi-1-specific siRNA compared to the nonspecific control (NC) (*P* < 0.05). Consistent with the inhibitory effects of Bmi-1-specific siRNA on glucose uptake, the relative lactate levels were significantly lower in AGS cells transfected with Bmi-1-specific siRNA vs the NC (*P* < 0.05) (Figure 2b).

### 3.3 Effects of Bmi-1 on GLUT1 mRNA and protein expression

**Figure 3:**
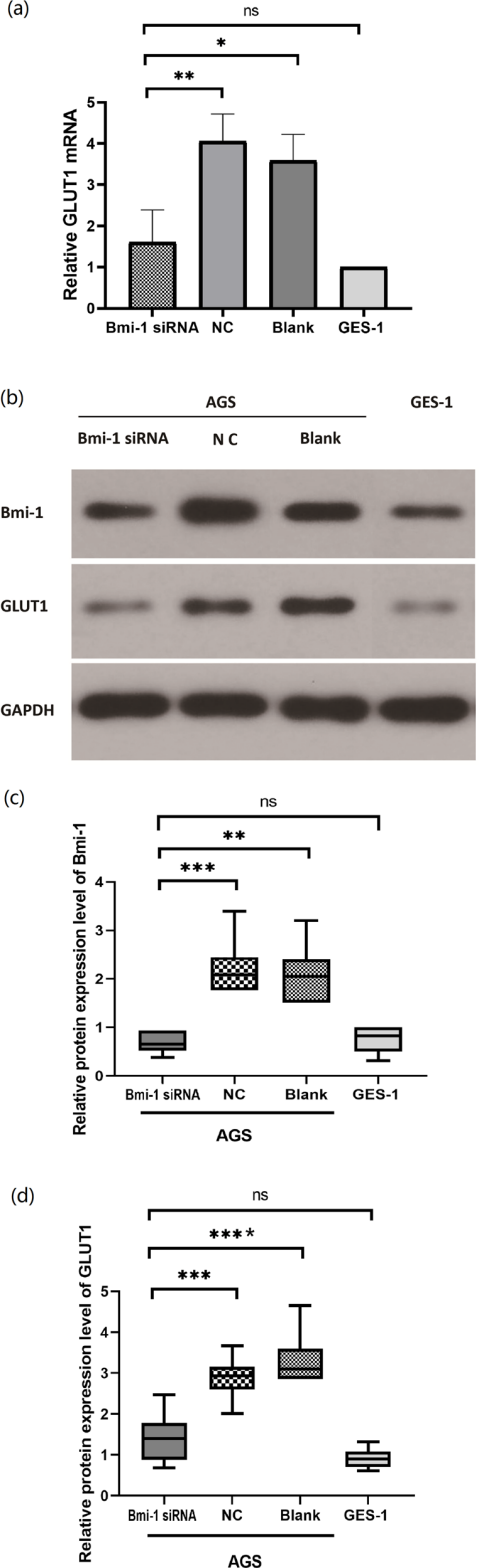
Effects of silencing Bmi-1 on GLUT1 protein expression in human gastric adenocarcinoma AGS cells. (a) qPCR analysis of GLUT1 mRNA expression levels in AGS cells transfected with Bmi-1-specific siRNA (Bmi-1 siRNA), nonspecific siRNA as a control (NC), with no treatment (Blank), as well as in human immortalized gastric epithelial GES-1 cells; (b) Western blot analysis of Bmi-1 and GLUT1 protein expression in AGS cells transfected with Bmi-1 siRNA, nonspecific siRNA as a control (NC), with no treatment (Blank), as well as in GES-1 cells; (c) quantification of the Bmi-1 protein levels shown in the western blots. The relative Bmi-1 protein expression was normalized to GAPDH. * indicates a statistical difference between the two groups, *P* < 0.05; (d) quantification of the GLUT1 protein levels shown in the western blots. The relative GLUT1 protein expression was normalized to GAPDH. * indicates a statistical difference between the two groups, *P* < 0.05.

Next we determined the effects of silencing Bmi-1 gene expression on GLUT1 in GAC by using AGS cells. AGS cells were transfected with Bmi-1-specific siRNA (Bmi-1 siRNA) or nonspecific siRNA as a control (NC). As shown in Figure 3a–c, Bmi-1 siRNA significantly suppressed the Bmi-1 protein expression compared with the NC in AGS cells. Notably, silencing Bmi-1 resulted in a marked reduction in GLUT1 mRNA and protein expression in AGS cells, and the difference was statistically significant between the Bmi-1 siRNA and NC groups (*P* < 0.05).

We also examined the Bmi-1 and GLUT1 protein levels in AGS cells and noncancerous human gastric epithelial GES-1 cells without any treatment. Western blot analysis revealed that both the Bmi-1 and GLUT1 protein levels were significantly higher in the AGS cells compared with the GES-1 cells (Figure 3b–d).

### 3.4 *In silico* analysis of putative binding sites for Bmi-1 in the promoter region of the GLUT1 gene

**Figure 4:**
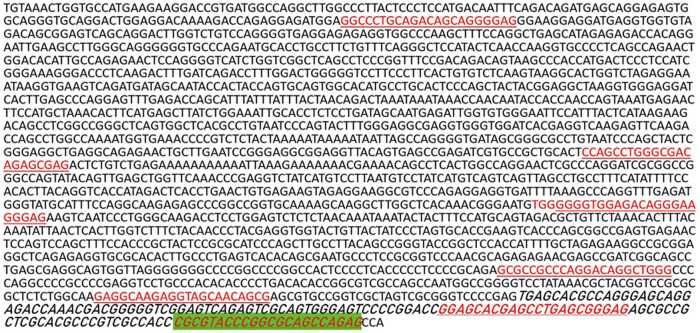
*In silico* analysis of putative binding sites for Bmi-1 in the promoter region of the GLUT1 gene. The hTFtarget database of human transcription factor targets (Hangzhou University of Science and Technology, Hanzhou, Zhejiang, China) was used for *in silico* analysis of putative binding sites for Bmi-1 in the promoter region of the GLUT1 gene spanning from 2,000 base pairs (bps) upstream to 200 bps downstream of the transcription start site. A total of seven putative binding sites were predicted and are denoted by red font. The one with the highest score for Bmi-1 binding is highlighted in green.

Considering that the transcription factor Bmi-1 could modulate the GLUT1 gene by binding to promoter or enhancer sequences, we conducted *in silico* analysis with hTFtarget, a free database of human transcription factor targets, that is available online (http://bioinfo.life.hust.edu.cn/hTFtarget#!/prediction) (Hanzhou University of Science and Technology, Hanzhou, Zhejiang, China). As illustrated in Figure 4, a number of putative binding sites for Bmi-1 were predicted in the promoter region of the GLUT1 gene spanning from 2,000 base pairs (bps) upstream to 200 bps downstream of its transcription start site. Of the seven putative binding sites identified (red font in Figure 4), one was predicted to have the highest potential for Bmi-1 binding (highlighted in green in Figure 4). These results suggest that the promoter region of the GLUT1 gene harbors putative binding sites for the transcription factor Bmi-1.

### 3.5 Interaction between the transcription factor Bmi-1 and the GLUT1 gene promoter region

**Figure 5:**
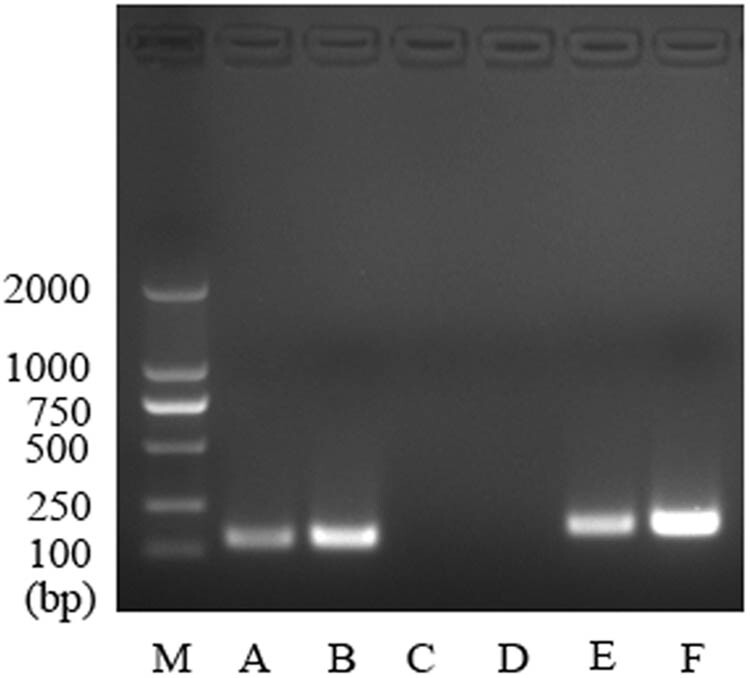
Chromatin immunoprecipitation (ChIP) assay of the interaction between the transcription factor Bmi-1 and the GLUT1 gene promoter region in AGS cells. A ChIP assay was conducted to examine the potential interaction between the transcription factor Bmi-1 and the GLUT1 promoter region. AGS cells were incubated with 1% formaldehyde for cross-linking. Chromatin was broken into fragments. Protein-DNA complexes were washed and disassociated, and the DNA disassociated with the protein-DNA complex was purified and analyzed by amplifying the target DNA using PCR. Lane M: standard DNA markers; Lane A: Bmi-1 antibody; Lane B: RT-input positive control; Lane C: negative control IgG; Lane D: RT-input negative control; Lane E: RNA polymerase II antibody and GAPDH primers in PCR; Lane F: RT-input positive control with GAPDH primers for PCR.

**Figure 6:**
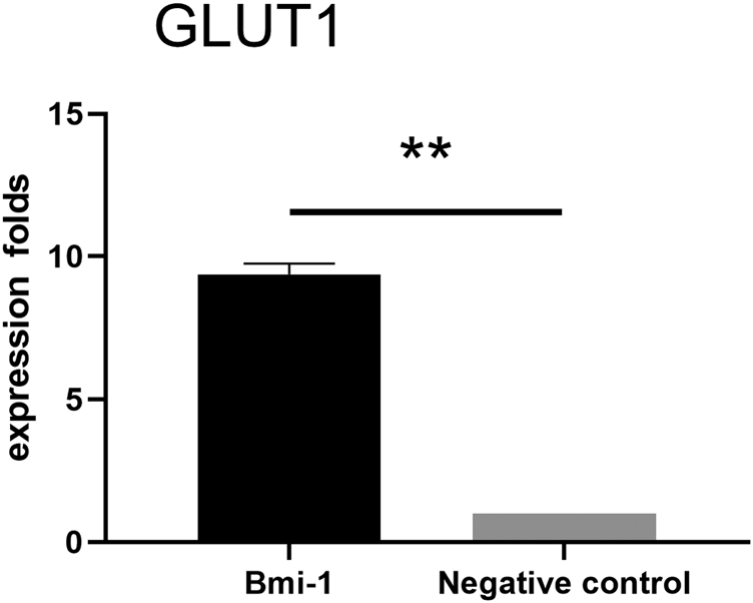
Amplification and quantification of the GLUT1 promoter region following the chromatin immunoprecipitation assay (ChIP). Real-time quantitative polymerase chain reaction (qPCR) was performed to amplify and quantify the GLUT1 promoter regions following ChIP analysis in the experimental group (Bmi-1-specific antibody) and the negative control group (IgG). ** indicates a statistical difference between the two groups, *P* < 0.001.

To determine if the putative binding sites in the GLUT1 promoter region for Bmi-1 could be functional, we performed ChIP assays to examine the potential interaction between the transcription factor Bmi-1 and the GLUT1 promoter region in AGS cells. As shown in Figure 5, the experimental group incubated with the Bmi-1-specific antibody showed the target PCR GLUT1 band (Lane A), which was not detected in the control group with IgG (Lane C). Following ChIP analysis, qPCR was performed to quantify the GLUT1 promoter region in the experimental group (Bmi-1-specific antibody) and negative control (IgG). As illustrated in Figure 6, the binding of Bmi-1 to the GLUT1 promoter region was markedly greater in the experimental group than in the negative control group (*P* < 0.001). The ChIP and ChIP-qPCR assays demonstrated that the transcription factor Bmi-1 directly interacted with the promoter region of the GLUT1 gene and thereby regulated its gene expression in AGS cells.

### 3.6 Effects of Bmi-1 on ^18^F-FDG uptake in nude mice bearing xenografts of AGS cells

**Figure 7:**
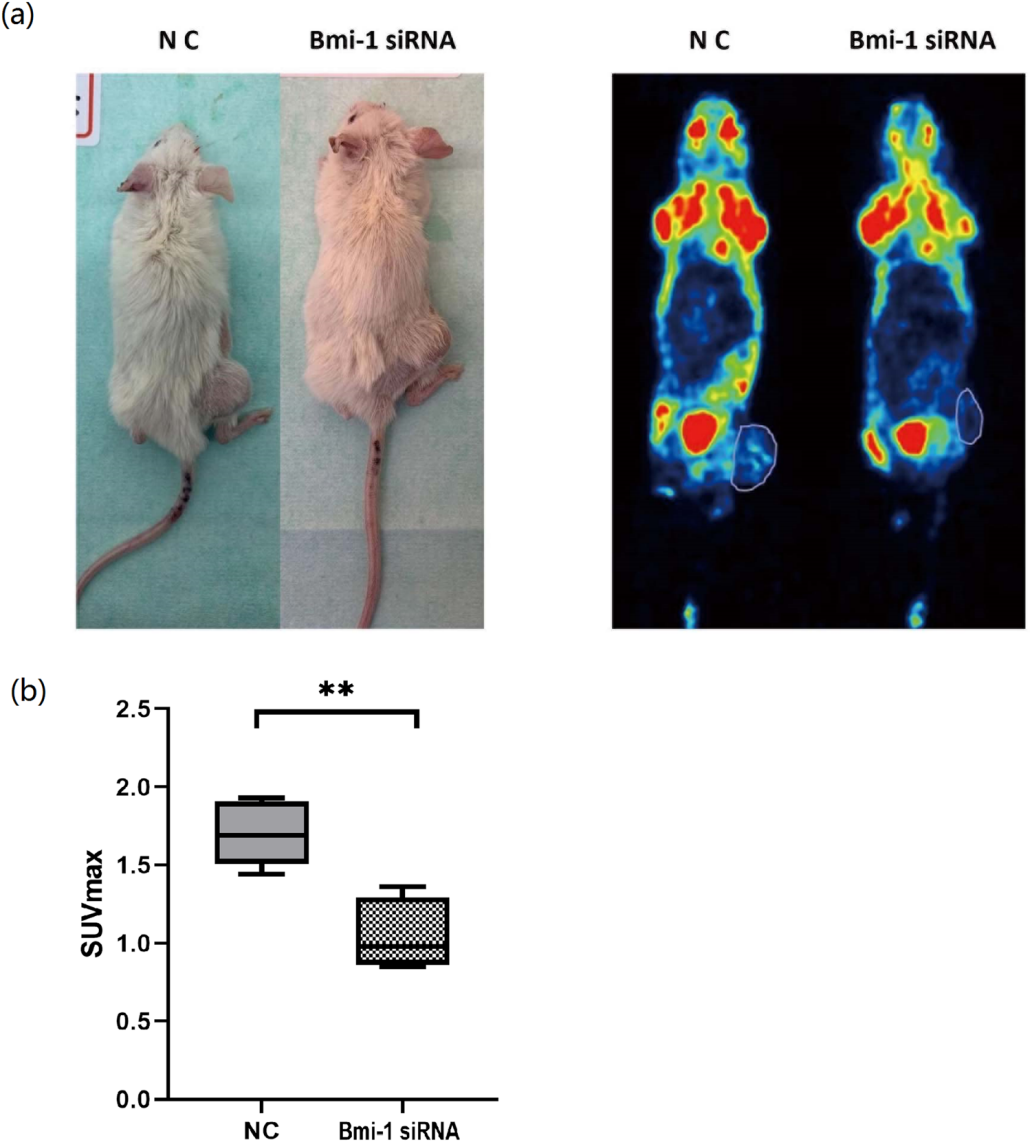
Effects of Bmi-1 on ^18^F-FDG uptake in nude mice bearing xenografts of gastric adenocarcinoma AGS cells. Nude mice bearing xenografts of AGS cells were treated with Bmi-1-specific siRNA in the experimental group (*n* = 5, 3 males and 2 females) or nonspecific siRNA (NC) in the control group (*n* = 5, 3 males and 2 females). (a) ^18^F-FDG PET/CT imaging of nude mice with xenografts of AGS cells treated with Bmi-1-specific siRNA or NC; (b) comparison of the SUV_max_ between the two groups of nude mice bearing xenografts of AGS cells treated with Bmi-1-specific siRNA or NC. ** indicates a statistical difference between the two groups, *P* < 0.05.

With evidence supporting a role for the transcription factor Bmi-1 in the regulation of GLUT1 gene expression and considering GLUT1 as a crucial uptake transporter of ^18^F-FDG in tumors, we hypothesized that the SUV_max_ acquired by noninvasive PET/CT imaging could be reduced in response to silencing of Bmi-1 gene expression. To test this hypothesis and to further determine the role of Bmi-1 in the regulation of GLUT1 in glucose metabolism, we performed PET/CT imaging in the two groups of nude mice bearing xenografts of AGS cells treated with Bmi-1-specific siRNA or NC as a control, respectively. As shown in Figure 7, ^18^F-FDG PET/CT imaging revealed xenografts in the experimental nude mice (Figure 7a), and the SUV_max_ was significantly lower in the mice bearing xenografts of AGS cells treated with Bmi-1-specific siRNA in comparison with the NC (*P* = 0.0015) (Figure 7b). These data indicate that the ^18^F-FDG uptake of AGS cells was decreased after the Bmi-1 gene expression was suppressed. This finding could be attributable to the downregulation of GLUT1 by Bmi-1. Furthermore, ^18^F-FDG PET/CT imaging holds potential to noninvasively evaluate the Bmi-1 gene expression and glucose metabolism of GAC.

## 4 Discussion

The present study on the potential role of Bmi-1 in the regulation of GLUT1 in GAC has the following major novel findings: (1) Both the Bmi-1 and GLUT1 protein levels were significantly elevated in GAC vs noncancerous tissues in patients (Figure 1) as well as in human GAC cells vs noncancerous human gastric epithelial cells (Figure 3); (2) A novel role of Bmi-1 in the modulation of glucose metabolism and GLUT1 at the transcriptional level was established by multiple lines of scientific evidence, including decrease in glucose uptake, lactate levels, and GLUT1 expression in response to silencing of Bmi-1 with its specific siRNA as well as *in silico* prediction of multiple binding sites for Bmi-1 in the promoter region of GLUT1 DNA, which was further evidenced by ChIP and ChIP-qPCR assays (Figures 2–6); (3) *In vivo* ^18^F-FDG PET/CT imaging studies in rodents bearing xenografts of AGS cells demonstrated that silencing of Bmi-1 led to a decrease in GLUT1 and glucose metabolism (Figure 7). These results indicate a direct interaction between the transcription factor Bmi-1 and the promoter region of GLUT1 DNA, showing that Bmi-1 plays a novel role in the upregulation of GLUT1 gene expression and glucose metabolism as well as in the enhancement of glucose uptake in GAC.

The Warburg effect, also referred to as the aerobic glycolysis of cancer cells, is a characteristic of glucose metabolism in cancer cells in which glycolysis occurs even under the conditions of sufficient oxygen to make energy for the excessive proliferation and other abnormal cellular processes of cancer cells. Mainly based on the Warburg effect of cancer cells, ^18^F-FDG PET/CT was developed as a useful noninvasive imaging technique to evaluate and manage malignant tumors, including the diagnosis, disease staging, and treatment planning and monitoring. It has been shown that GLUT1 plays an important role in glucose uptake in cancer cells, which is a rate-limiting step in glucose metabolism [5,6]. In fact, extensive previous studies have shown that GLUT1 gene expression is markedly elevated in various cancers, including gastric cancer, and that higher levels of GLUT1 are correlated with a higher proliferation rate of cancer cells, more aggressive progression, and a poorer prognosis of cancer patients [7,8,13,14]. Therefore, GLUT1 is considered as the most important factor in determining FDG uptake [14]. To date, the molecular mechanisms underlying the regulation of GLUT1 in cancer cells are unclear. Many studies have noted that a number of oncogenes and growth factors affect the gene expression and activity of GLUT1 [15,16], which have an impact on the glucose metabolism of cancer cells [17].

More recently, we identified a positive correlation between GLUT1 expression and Bmi-1 expression in gastric cancer, and a higher expression of Bmi-1 was associated with a higher degree of tumor malignancy (data not published yet). Some previous studies by others have also indicated that Bmi-1 expression is increased in GAC and that the overexpression of Bmi-1 leads to enhancement in the proliferation, invasion, and metastasis of gastric cancer [9,10,11,12]. Several studies of patients with gastric cancer have suggested that Bmi-1 may serve as an independent prognostic factor in predicting the survival of patients with gastric cancer [18,19,20]. Notably, Bmi-1 in combination with C-X-C motif chemokine receptor 4 presented a significantly higher sensitivity and selectivity compared with carcinoembryonic antigen and cancer antigen 19-9 [21]. Despite the Warburg effect in malignant cells and other previous findings of the involvement of Bmi-1 in tumor progression and metastasis [22,23,24,25,26], no studies have reported on the relationship between Bmi-1 and GLUT1 in cancer cells, including GAC cells or tissues [27].

The present study was the first attempt to examine the potential regulatory role of Bmi-1 on the expression of GLUT1 in GAC cell cultures and mice. We found that the Bmi-1 and GLUT1 protein expressions were significantly higher in AGS cells than in normal human gastric epithelial GES-1 cells. In addition, silencing of Bmi-1 led to a significant reduction in GLUT1 expression in cell cultures. Moreover, the functionality of Bmi-1 in the regulation of GLUT1 gene expression was demonstrated by multiple lines of evidence, including initial *in silico* analysis identifying seven putative binding sites for the transcription factor Bmi-1 in the promoter region of the GLUT1 gene, by the expected effects of Bmi-1-specfic siRNA on the GLUT mRNA levels and glucose uptake, and by ChIP assays showing the direct interaction between the transcription factor Bmi-1 and the GLUT1 promoter region. Therefore, our findings increase the understanding of the role that the transcription factor Bmi-1 plays in the regulation of target gene expression [27]. Our results, together with those of others, suggest that Bmi-1 could be used as a new target in the development of new therapies for gastric cancer [28,29,30].

It merits attention that a further *in vivo* investigation with ^18^F-FDG PET/CT imaging in nude mice bearing xenografts of AGS cells showed that silencing of Bmi-1 resulted in a significant reduction in SUV_max_, an important FDG uptake parameter as measured by PET/CT imaging. These results further support the novel regulatory role for the transcription factor Bmi-1 in regulating GLUT1 expression at the transcriptional level in gastric cancer.

In conclusion, the findings of this study demonstrated that Bmi-1 directly binds to the promoter region of GLUT1, which upregulates GLUT1 gene expression at the transcriptional level and therefore is involved in glucose metabolism and the enhancement of glucose uptake in GAC. These results also implicate that noninvasive ^18^F-FDG PET/CT imaging could be used for the evaluation of Bmi-1 and glucose uptake in GAC.
